# Supplementary material for: Circulating MicroRNA: Incident Asthma Prediction and Vitamin D Effect Modification
Source: J Pers Med. 2021 Apr 16;11(4):307. doi: 10.3390/jpm11040307 (PMC8073146; doi:10.3390/jpm11040307)

Circulating MicroRNA: Incident Asthma Prediction and Vitamin D Effect Modification

Jiang Li[1,2], Anshul Tiwari[2], Hooman Mirzakhani[2], Alberta L Wang[2], Alvin T Kho[2,3], Michael J McGeachie[2], Augusto A. Litonjua[4], Scott T Weiss[2], Kelan G Tantisira[2,5,*]

1 Research Center, The Seventh Affiliated Hospital of Sun Yat-Sen University, Shenzhen, Guangdong 518107, China;
2 Channing Division of Network Medicine, Brigham and Women's Hospital and Harvard Medical School, Boston, MA 02115, USA;
3 Computational Health Informatics Program, Boston Children's Hospital, Boston, MA 02115, USA
4 Division of Pediatric Pulmonary Medicine, Golisano Children's Hospital at Strong, University of Rochester Medical Center, Rochester, NY 14642, USA;
5 Division of Pediatric Respiratory Medicine, University of California San Diego and Rady Children's Hospital, San Diego, CA 92123, USA;
* Correspondence: ktantisira@health.ucsd.edu; Tel.: +1-(858)-966-5846; Fax: +1-(858)-966-8457

Supplemental Tables

Table S1. Significant miRNAs in Main Analysis.

miRNA	VDAART			Project Viva		
		95% CI for OR			95% CI for OR	
	OR	Lower Bound	Upper Bound	OR	Lower Bound	Upper Bound
hsa-miR-548k	0.398	0.205	0.772	0.667	0.212	2.099
hsa-miR-6509-5p	2.582	1.226	5.438	---	---	---
hsa-miR-21-5p	0.27	0.096	0.764	1.561	0.362	6.723
hsa-miR-30d-3p	0.461	0.236	0.898	---	---	---
hsa-miR-195-5p	1.823	1.061	3.131	---	---	---
hsa-miR-505-3p	2.502	1.088	5.755	0.961	0.375	2.461

Table S2. Stratified analysis in Placebo Group.

miRNA	OR	95% CI for OR	
		Lower Bound	Upper Bound
hsa-miR-505-3p	7.83	1.78	34.34
hsa-miR-193b-5p	0.51	0.29	0.92
hsa-miR-7-1-3p	0.26	0.07	0.90
hsa-miR-340-3p	2.00	1.05	3.82
hsa-miR-5010-5p	0.45	0.21	0.97
hsa-miR-548k	0.43	0.19	0.99

Table S3. Validated significant miRNAs in Project Viva.

miRNA	VDAART						Project Viva					
	High			Interaction			High			Interaction		
	OR	Lower Bound	Upper Bound	OR	Lower Bound	Upper Bound	OR	Lower Bound	Upper Bound	OR	Lower Bound	Upper Bound
hsa-miR-574-5p	7.20	1.16	44.78	19.20	2.38	155.11	27.59	0.19	3995.22	3.90	0.15	103.06
hsa-miR-151a-5p	0.15	0.02	0.86	0.05	0.01	0.42	0.34	0.02	4.77	0.37	0.01	12.54
hsa-miR-125b-2-3p	5.46	1.38	21.52	8.62	1.77	41.84	2.90	0.19	44.37	35.01	0.12	10403.99
hsa-miR-6852-5p	0.19	0.05	0.79	0.12	0.03	0.58	0.21	0.02	1.75	0.32	0.03	3.59
hsa-miR-342-3p	3.72	1.31	10.62	5.83	1.63	20.89	2.71	0.51	14.38	3.38	0.10	114.40
hsa-miR-370-3p	0.33	0.13	0.83	0.33	0.12	0.94	0.51	0.18	1.44	0.86	0.12	6.21
hsa-miR-193b-5p	2.85	1.23	6.60	5.55	1.99	15.45	1.82	0.51	6.49	3.10	0.43	22.47
hsa-miR-122-5p	2.68	1.21	5.96	3.05	1.20	7.74	1.75	0.60	5.10	3.60	0.70	18.54
hsa-miR-215-5p	2.62	1.11	6.17	3.05	1.14	8.13	4.67	0.63	34.38	38.44	0.36	4150.60

Table S4. KEGG Enrichment Analysis of Target Genes of Validated miRNAs.

Name	Hits	P Value	FDR
Chronic myeloid leukemia	30	4.41E-09	4.41E-07
Pathways in cancer	78	1.04E-08	4.80E-07
Cell cycle	41	1.44E-08	4.80E-07
Colorectal cancer	22	7.91E-08	1.98E-06
Pancreatic cancer	25	1.52E-06	3.04E-05
TGF-beta signaling pathway	28	2.47E-06	4.12E-05
Prostate cancer	28	5.39E-06	7.70E-05
HTLV-I infection	49	1.31E-05	1.64E-04
ErbB signaling pathway	27	1.69E-05	1.88E-04
Glioma	21	7.60E-05	7.60E-04
Wnt signaling pathway	36	1.35E-04	1.23E-03
Melanoma	21	1.58E-04	1.32E-03
Bladder cancer	12	2.00E-04	1.45E-03
Jak-STAT signaling pathway	27	2.03E-04	1.45E-03
Small cell lung cancer	23	2.57E-04	1.71E-03
Focal adhesion	45	2.93E-04	1.83E-03
Neurotrophin signaling pathway	30	7.53E-04	4.28E-03
Epstein-Barr virus infection	24	7.71E-04	4.28E-03
Osteoclast differentiation	29	9.31E-04	4.90E-03
Non-small cell lung cancer	16	9.85E-04	4.90E-03
Regulation of actin cytoskeleton	40	1.03E-03	4.90E-03
p53 signaling pathway	19	1.27E-03	5.77E-03
Renal cell carcinoma	17	1.91E-03	8.30E-03
Epithelial cell signaling in Helicobacter pylori infection	12	2.54E-03	1.06E-02
Sphingolipid metabolism	13	6.49E-03	2.60E-02
Acute myeloid leukemia	15	7.38E-03	2.74E-02
Pyrimidine metabolism	23	7.45E-03	2.74E-02
Pertussis	14	7.68E-03	2.74E-02
Insulin signaling pathway	29	8.49E-03	2.93E-02
Thyroid cancer	9	9.21E-03	3.07E-02
Herpes simplex infection	23	9.51E-03	3.07E-02
T cell receptor signaling pathway	22	1.04E-02	3.18E-02
Adherens junction	17	1.05E-02	3.18E-02
Endometrial cancer	12	1.18E-02	3.47E-02
Protein processing in endoplasmic reticulum	27	1.26E-02	3.60E-02
Apoptosis	19	1.35E-02	3.75E-02
Chagas disease (American trypanosomiasis)	20	1.40E-02	3.78E-02
Measles	22	1.65E-02	4.34E-02
Toll-like receptor signaling pathway	21	1.81E-02	4.55E-02
Viral myocarditis	8	1.82E-02	4.55E-02
MAPK signaling pathway	48	1.92E-02	4.68E-02
Fc epsilon RI signaling pathway	17	2.07E-02	4.93E-02

Supplemental Figures

Figure S1. Boxplot of normalized miRNA counts of hsa-miR-574-5p and hsa-miR-151a-5p. (A) Boxplot of hsa-miR-574-5p in high vitamin D treatment group. B) Boxplot of hsa-miR-151a-5p in high vitamin D treatment group.

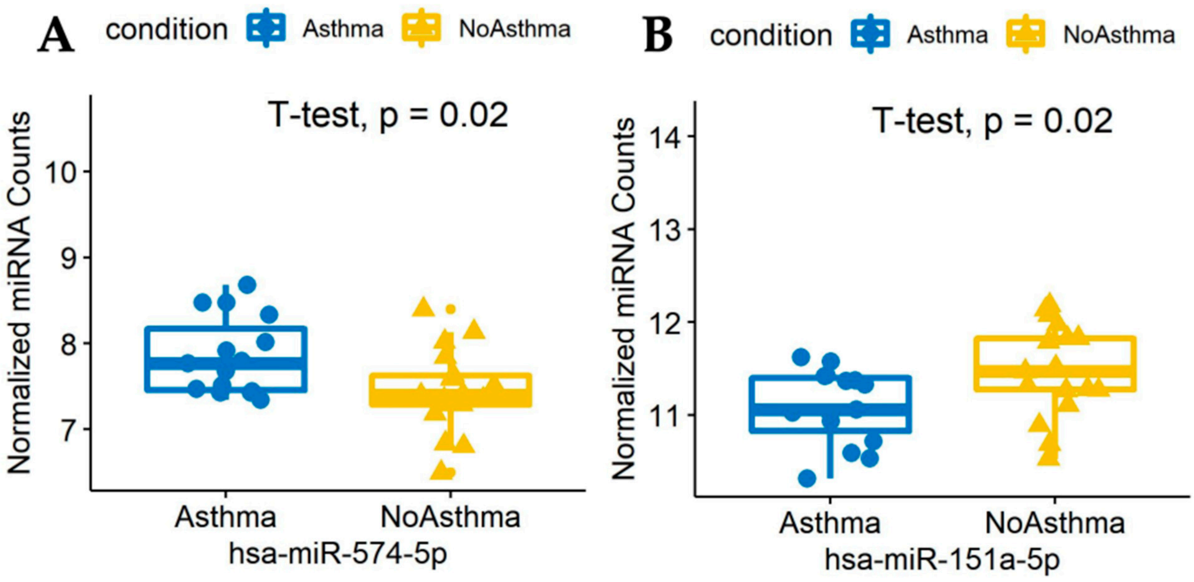

Figure S2. Meta-analysis of the effect modification for 9 validated miRNAs.

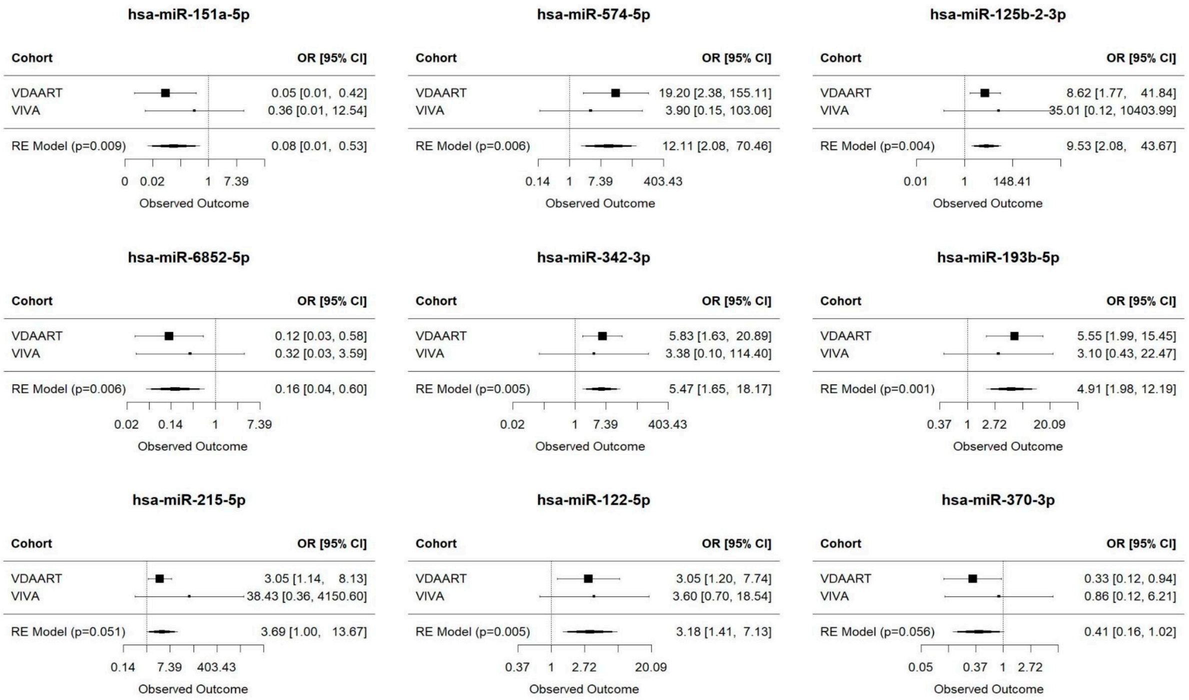

Supplement: Supplementary file 1 [file jpm-11-00307-s001.zip › jpm-1163359-supplementary.pdf]
